# Accessory Auricle (Polyotia) in a Pediatric Patient: A Rare Congenital Anomaly

**DOI:** 10.7759/cureus.108900

**Published:** 2026-05-15

**Authors:** Nilesh Khakholia, Gargee Gogoi, Barnakshi Deka

**Affiliations:** 1 Pediatrics, Gauhati Medical College and Hospital, Guwahati, IND; 2 Oral and Maxillofacial Surgery, Regional Dental College, Guwahati, IND

**Keywords:** accessory auricle, auricular hillocks, external ear malformation, pediatric congenital anomaly, polyotia

## Abstract

Accessory auricle and polyotia are rare congenital anomalies of the external ear resulting from abnormal development of the auricular hillocks. Polyotia represents a severe variant in which the accessory structure resembles an additional pinna. We report a paediatric case of unilateral accessory auricle in a child presenting to the Department of Pediatrics at Gauhati Medical College and Hospital. Clinical evaluation revealed no associated systemic abnormalities. The patient underwent surgical excision by the Department of ENT with satisfactory aesthetic outcome and no postoperative complications. The present case is of particular educational value because isolated unilateral accessory auricle without syndromic association is exceedingly uncommon and may pose a diagnostic challenge due to its resemblance to other congenital auricular malformations. This report emphasizes the importance of differentiating the accessory auricle from true polyotia and other external ear anomalies through careful clinical assessment. It also highlights the role of multidisciplinary evaluation to exclude associated craniofacial, auditory, and systemic abnormalities, which are frequently reported in such congenital conditions. Furthermore, the case demonstrates that timely surgical management can achieve favourable cosmetic outcomes with minimal morbidity, thereby contributing to the limited literature on rare auricular developmental anomalies in the paediatric population.

## Introduction

Congenital anomalies of the external ear encompass a wide spectrum ranging from minor preauricular tags to severe deformities such as microtia and polyotia [1]. These anomalies arise due to defective development and fusion of the six auricular hillocks derived from the first and second branchial arches during embryogenesis. The first three hillocks, originating from the first branchial arch, contribute primarily to the formation of the tragus and anterior auricle, whereas the remaining three hillocks from the second branchial arch form the helix, antihelix, antitragus, and lobule. Abnormal migration, duplication, or incomplete fusion of these hillocks may result in congenital auricular malformations such as an accessory auricle and polyotia [1,2].

Polyotia is an extremely rare condition characterized by an accessory auricle that resembles a normal pinna rather than a rudimentary appendage [1,3]. The reported incidence is very low, with fewer than 30 cases described in the literature. Reports from the Indian subcontinent are particularly limited, with only a few isolated cases documented from India [1,3]. Although it may occur as an isolated anomaly, it has also been associated with craniofacial syndromes such as Goldenhar syndrome and Treacher Collins syndrome [2,4].

Early diagnosis and appropriate surgical intervention are essential, particularly in paediatric patients, to achieve optimal cosmetic outcomes and reduce psychosocial impact.

## Case presentation

A nine-year-old female patient presented to the Department of Pediatrics at Gauhati Medical College and Hospital, Guwahati, with a congenital swelling over the left preauricular region, present since birth.

**Figure 1:**
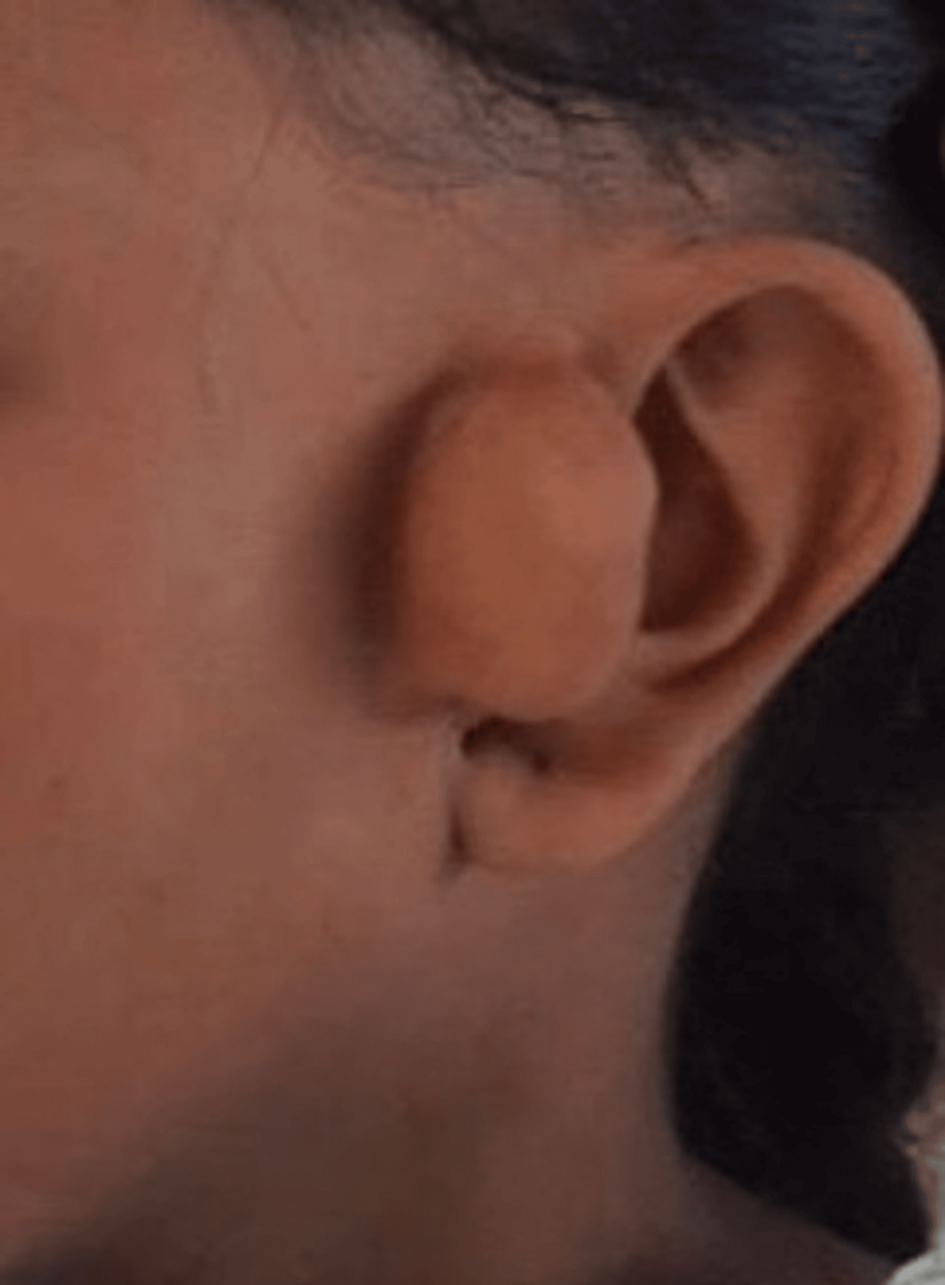
Preoperative clinical presentation

On clinical examination, a well-defined accessory auricular structure measuring approximately 4 cm x 3 cm was noted anterior to the normal auricle (Figure 1).

The lesion consisted of skin with an underlying cartilaginous component. The native auricle was well formed, and the external auditory canal was patent. There was no discharge, tenderness, or signs of infection. On palpation, the lesion was soft, mobile, and non-fixed to the underlying structures.

No similar family history was reported. General physical and systemic examination was unremarkable. Clinical hearing assessment was normal, and audiometry and ultrasound of the abdomen did not reveal any associated anomalies.

The patient was referred to the Department of ENT at Gauhati Medical College and Hospital for surgical management.

**Figure 2:**
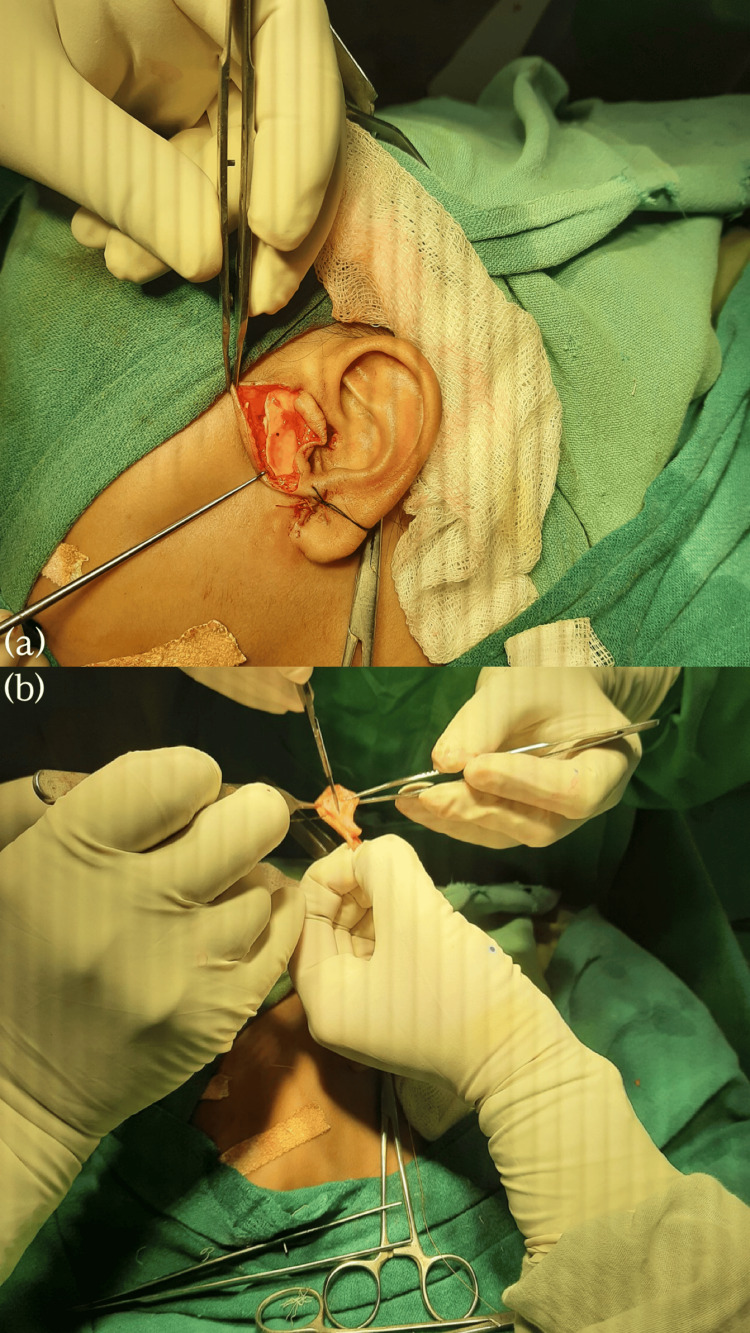
Intra-operative procedure (a) Surgical exposure, (b) excision in toto

Informed written consent was taken from the parents. Under general anesthesia, complete excision of the accessory auricle was performed. Preoperative markings were made using the contralateral ear as a reference. An incision was placed along the natural contour, followed by careful dissection to expose and excise the cartilaginous component (Figures 2a, 2b).

**Figure 3:**
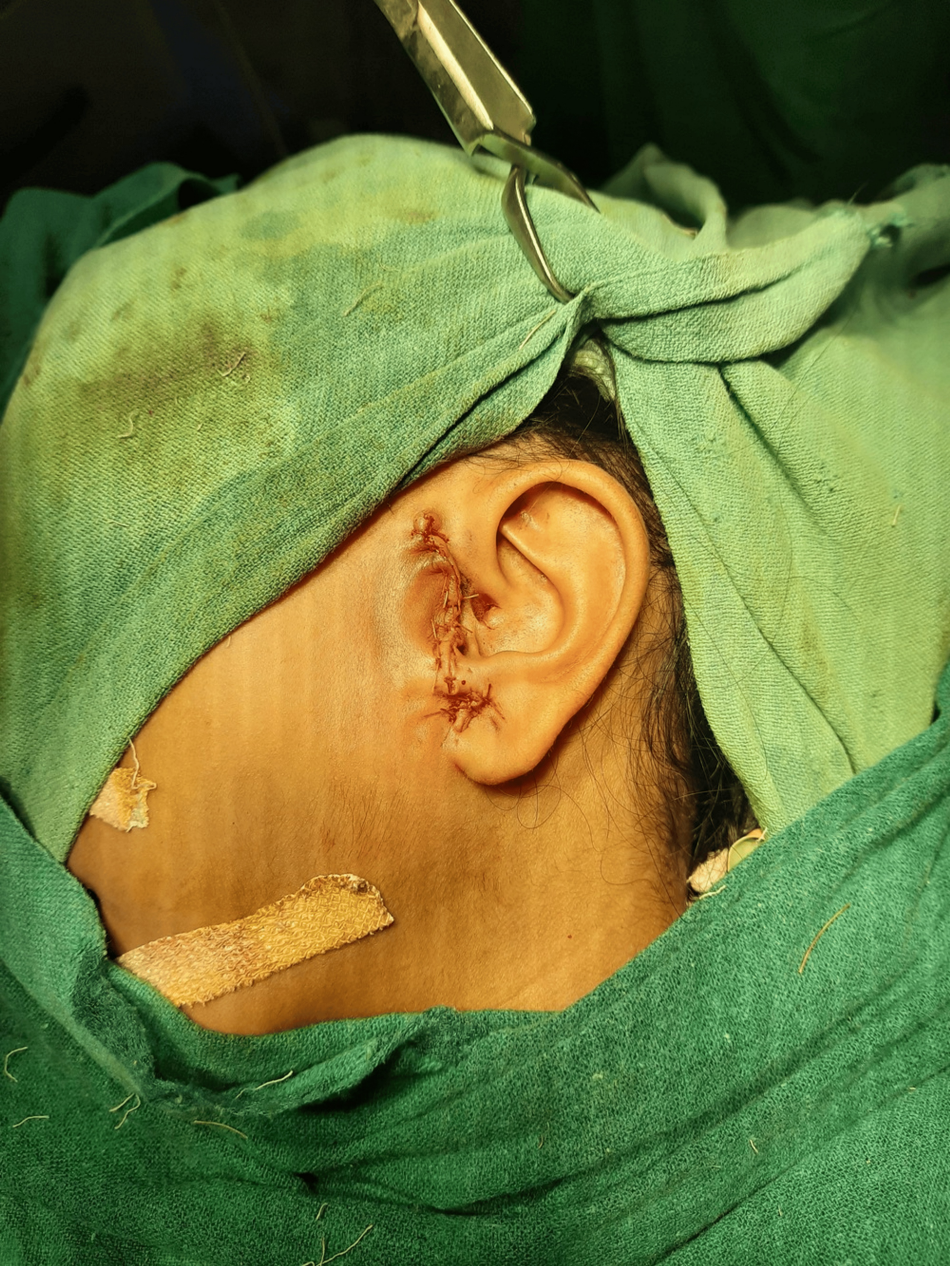
Closure of the defect using 4-0 Ethilon (Ethicon, Raritan, NJ, USA)

Redundant skin was trimmed, and the wound was closed in layers using 4-0 Ethilon (Ethicon, Raritan, NJ, USA), with care taken to preserve adjacent structures, including the facial nerve (Figure 3).

**Figure 4:**
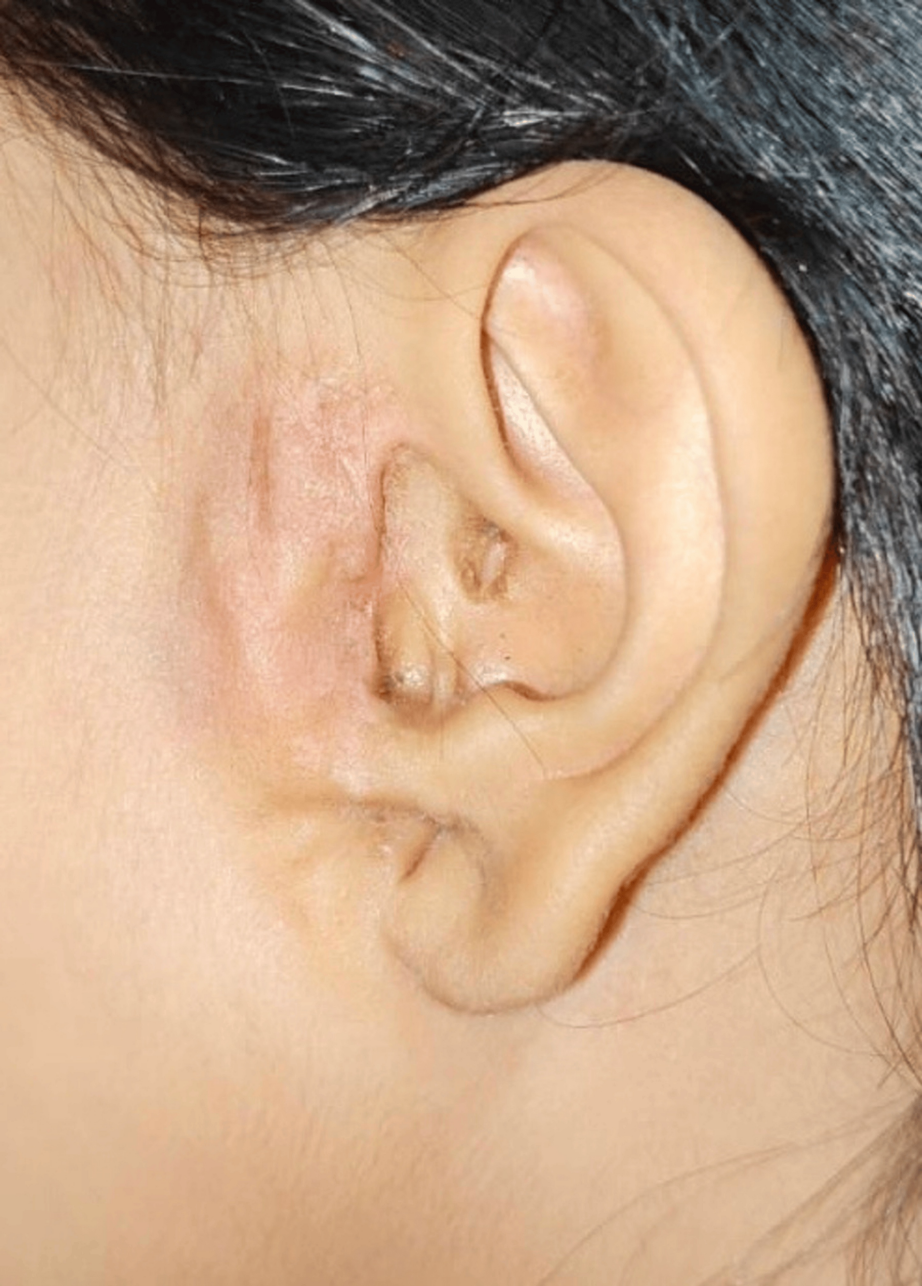
Satisfactory postoperative healing

The postoperative period was uneventful. No complications such as infection or facial nerve deficit were observed. At follow-up after three months, the patient demonstrated satisfactory healing with a good cosmetic outcome and no recurrence (Figure 4).

## Discussion

Accessory auricular anomalies result from aberrations in the development of the auricular hillocks during embryogenesis [1,2]. The auricle develops from six mesenchymal proliferations arising from the first and second branchial arches, and failure of proper fusion or migration can lead to accessory auricular formations [1].

Polyotia is a rare and more extensive form of accessory auricle, in which the accessory structure resembles a duplicated pinna [1,3]. The condition is extremely uncommon, with limited cases reported in the literature [3]. It is most often unilateral and may occur either as an isolated anomaly or as part of a syndrome [2,4]. Clinically, polyotia should be differentiated from other congenital auricular anomalies such as accessory tragus, preauricular tag, auricular appendage, microtia with redundant tissue, and branchial arch remnants, as these lesions may present with similar external features but differ in embryological origin and management.

In the present case, the anomaly was isolated, with no associated systemic findings. This is consistent with previous reports suggesting that accessory auricular anomalies are frequently isolated and may follow an autosomal dominant pattern of inheritance in some cases [5]. However, due to possible associations with craniofacial syndromes and renal anomalies, comprehensive evaluation including audiological assessment and renal imaging is recommended [2,4].

Previously reported cases of polyotia have described variable clinical presentations ranging from small accessory auricular appendages to well-formed duplicated pinna-like structures associated with craniofacial syndromes, hearing abnormalities, and first or second branchial arch defects [3,6,7]. Most reported cases have been unilateral, similar to the present case; however, several authors have documented associated anomalies necessitating extensive systemic evaluation and long-term follow-up [2,4]. In contrast, our patient presented with an isolated unilateral accessory auricle without any associated auditory, renal, or craniofacial abnormalities, thereby representing a relatively uncommon non-syndromic presentation.

Furthermore, unlike previously reported cases requiring complex auricular reconstruction or conchal correction to restore facial symmetry [6,7], the present case was managed successfully with simple surgical excision due to the absence of significant cartilaginous deformity or conchal involvement. The excellent postoperative aesthetic outcome and absence of complications in our patient are consistent with earlier studies suggesting that early intervention in isolated lesions is associated with favourable cosmetic and psychosocial outcomes [3,4].

Surgical excision remains the definitive treatment for accessory auricle and polyotia. The primary goals include complete removal of accessory cartilage, preservation of adjacent structures such as the facial nerve, and restoration of symmetry [1,7]. Konaş et al. outlined key surgical principles, including excision of accessory cartilage, trimming of redundant skin, and careful reconstruction to achieve optimal aesthetic outcomes [7].

Early surgical intervention in paediatric patients is advantageous, as it minimizes psychological distress and improves social acceptance. Literature supports early correction to achieve better aesthetic and functional outcomes [3,4].

## Conclusions

Accessory auricular anomalies are rare congenital malformations that require careful clinical evaluation to exclude associated systemic and craniofacial abnormalities. The present case highlights the importance of detailed morphological assessment and appropriate differential diagnosis in patients presenting with congenital auricular deformities. In our patient, surgical excision resulted in a satisfactory postoperative aesthetic outcome without complications during follow-up. A multidisciplinary approach involving paediatricians, otorhinolaryngologists, plastic or reconstructive surgeons, radiologists, and audiologists is valuable for comprehensive evaluation and individualized management of such rare congenital auricular anomalies.
